# Putative Role of a Yet Uncharacterized Protein Elicitor PeBb1 Derived from *Beauveria bassiana* ARSEF 2860 Strain against *Myzus persicae* (Homoptera: Aphididae) in *Brassica rapa* ssp. *pekinensis*

**DOI:** 10.3390/pathogens9020111

**Published:** 2020-02-11

**Authors:** Talha Nazir, Abdul Hanan, Abdul Basit, Muhammad Zeeshan Majeed, Tauqir Anwar, Iqra Nawaz, Dewen Qiu

**Affiliations:** 1 State Key Laboratory for Biology of Plant Diseases and Insect Pests, Institute of Plant Protection, Chinese Academy of Agricultural Sciences, Beijing 100081, China; talha23december@gmail.com (T.N.); hunnyuos@gmail.com (A.H.); malikbasituaf@gmail.com (A.B.); 2 Department of Entomology, College of Agriculture, University of Sargodha, Sargodha 40100, Pakistan; zeeshan.majeed@uos.edu.pk; 3 Department of Entomology, University of Agriculture, Faisalabad 38000, Pakistan; tauqeer26@gmail.com; 4 Research Institute of Pomology, Chinese Academy of Agricultural Science, Ministry of Agriculture, Xingcheng 125100, China; iqranawazcaas@gmail.com

**Keywords:** *Beauveria bassiana*, elicitor protein, induced systemic resistance, *Myzus persicae*, fecundity, jasmonic acid pathway, ethylene pathway

## Abstract

This study reports the characterization of protein elicitor PeBb1 derived from entomopathogenic fungus *Beauveria bassiana* ARSEF-2860 strain and its putative role in induced systemic resistance in *Brassica rapa* ssp. *pekinensis* against green peach aphid *Myzus persicae*. The sequence of purified elicitor protein was matched with the genomic sequence of a hypothetical protein BBA_10269 from *B. bassiana* ARSEF-2860 (GenBank Accession No. XP_008603588.1). The protein-encoding gene *PeBb1* contained 534 bp cDNA encoding a polypeptide of 177 amino acids with a molecular mass of 19 kDa. The recombinant elicitor protein was expressed in *Escherichia coli* using pET-28a (+) expression vector and induced necrosis in the leaves of tobacco. The effects of elicitor protein on aphid *M. persicae* was determined by applying three different concentrations of PeBb1 (i.e., 26, 35, 53 μM) on *B. rapa* plants at 4-leaf stage and the treated plants were exposed to newly emerged (0–6 h old) apterous adult aphids. Bioassay results showed significant (*p* < 0.05) sub-lethal effects of the exogenous application of PeBb1 elicitor on *M. persicae*. Moreover, the RT-qPCR gene expression analyses showed a significant up-regulation of most of the key genes linked to ethylene (ET)- and jasmonic acid (JA)-associated plant defense pathways in elicitor-treated plants. These results not only recommend the putative utilization of PeBb1 elicitor protein in future biological pest control strategies against phloem-feeding insect pests such as *M. persicae*, but also help in better comprehension of the mechanisms through which beneficial fungi trigger the induced plant resistance.

## 1. Introduction

Phytophagous insect pests are one of the major threats to agricultural production across the globe. These pests are estimated to cause more than 15% loss to global crop production annually [1,2]. Contemporary pest management programs against these insects primarily rely on the application of synthetic chemical insecticides that have always been an inevitable part of plant protection strategies [3]. Widespread and irrational use of these synthetic chemicals however has led to various health and environmental issues such as the eradication of non-target species including insect predators and parasitoids, contamination of soil, air and water resources, pest resistance and resurgence [4]. Increasing ill-effects being manifested by the conventional synthetic insecticides necessitate looking for the alternate biorational pest control tactics such as microbial biopesticides [5]. Many microbes including entomopathogenic fungi, bacteria, viruses, and nematodes have shown effectiveness against a large number of insect pest species [6,7]. With more than 750 species worldwide, entomopathogenic fungi provide a promising potential for biological control of insect pests. Particularly, fungal species belonging to genera Beauveria, Isaria, Lecanicillium, and Metarhizium exhibit an excellent pathogenicity against many insect pests [8,9,10].

Because of their low mammalian toxicity and residual activity and high host specificity [11], entomopathogenic fungi have been successfully employed against a number of agricultural, urban, and medically important insect pests [12,13,14]. However, the delayed pest mortality by entomopathogenic fungi as compared to synthetic insecticides can be improved either by increasing their toxicity through genetic modifications or by identifying and developing their bioactive metabolites exhibiting lethal and sublethal toxicity to insect pests [15,16,17,18]. Many entomopathogenic fungi have been reported to secrete different antifeedant, insecticidal, and toxic bioactive substances in the broth cultures [19,20,21].

Moreover, many entomopathogenic fungal strains can develop endophytically inside the plant tissues and induce systemic resistance against various abiotic and biotic stresses including herbivores and pathogens [22,23]. Recently, certain elicitor proteins derived from pathogenic fungi have been demonstrated to evoke induced systemic resistance and defense response in different plant species against various pathogenic organisms and phytophagous insect pests [23,24,25]. Plants develop resistance to their prospective attackers (pathogens and insect pests) at early stages through the induction of plant immune system [26,27,28]. In defense response, all signal molecules might be involved and could regulate and control the downstream signaling pathways through metabolic changes and gene expression [29]. In response to attack by pathogens and insect pests, plant defense systems are usually regulated by multiple signaling pathways including three key signaling molecules i.e., JA, SA, and ET [30,31,32].

This laboratory study was aimed to extract and purify an elicitor protein molecule from an entomopathogenic fungus *Beauveria bassiana sensu lato*, strain ARSEF 2860, followed by its molecular characterization and potential bioactivity against green peach aphid *Myzus persicae* Sulzer on Chinese cabbage (*Brassica rapa* ssp. *pekinensis*) plants. Moreover, the expression levels of important genes linked with ethylene (ET) and jasmonic acid (JA) plant defense pathways were measured by RT-qPCR in order to elucidate the mechanism of any local or systemic resistance induced by recombinant fungal protein in *B. rapa* plants against *M. persicae*. This research will elucidate the potential role of microbe-associated molecular patterns (MAMPs)-type protein elicitor derived from entomopathogenic fungi as novel microbial pest management tool and will enhance our understanding toward the possibility for the development of more virulent strains of entomopathogenic fungi through genetic improvements.

## 2. Results

### 2.1. Purification, Identification, and Characterization of PeBb1 Protein

**Figure 1:**
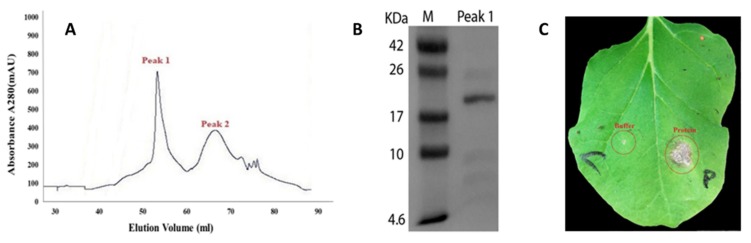
Purification of PeBb1 protein from *B. bassiana* strain ARSEF 2860 (BB-72). (**A**) Ion-exchange chromatography produced two peaks (1 and 2) of crude protein obtained from the ammonium sulfate precipitation and purified by ÄKTA Explorer 10 protein purification system; (**B**) PeBb1 (Peak 1) resolved on a SDS-PAGE gel; (**C**) necrosis induced by PeBb1 (53 µM) in tobacco leaves recorded at 24 h post infiltration. Control side of leaf lamina was treated with mock buffer (50 mM Tris-HCL, pH 8.0).

The crude protein from *B. bassiana* strain ARSEF 2860 (BB-72) was dialyzed and anion exchange chromatography was carried out and finally proteins were eluted with buffer B. Two protein peaks (Figure 1A) were collected, desalted, and injected into the leaves of tobacco (*Nicotiana tabacum* cv. Samsun-NN) to determine the activity of elicitor. Thereafter, we ran SDS-PAGE gel and the results revealed a single band of molecular weight of 19 kDa (Figure 1B). This protein was named as PeBb1. The band was recovered and bioassayed for necrosis-inducing activity (Figure 1C). SDS-PAGE was again subjected to mass spectrometry analysis. In brief, a single protein band was cut from SDS-PAGE gel for the recognition through liquid chromatography-mass spectrometry analysis. The outcomes were investigated by MASCOT software (Matrix Science Inc., London, UK) and we acquired the most matching protein (i.e., BBA_10269 hypothetical protein, GenBank: EJP60782.1). 

### 2.2. Gene Cloning

**Figure 2:**
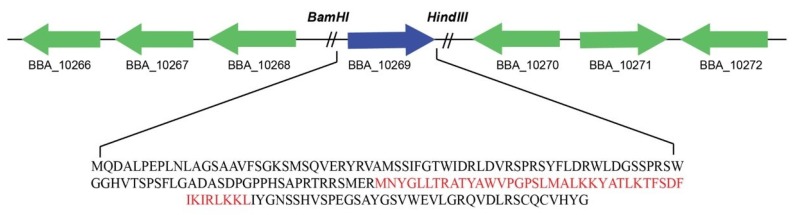
Schematic representation showing the gene BBA_10269 location in the genome of *Beauveria bassiana*. The red portion in the protein sequence shows active binding domain inside protein.

**Figure 3:**
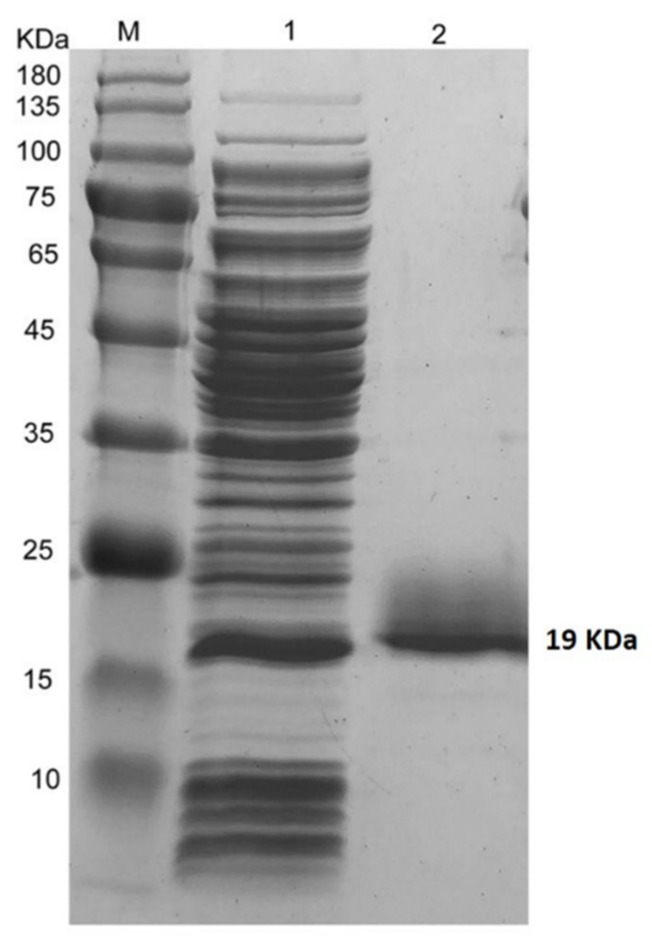
Purification of recombinant PeBb1 protein. 1 = total *E. coli* expressed proteins, 2 = purified His-tagged PeBb1 protein, M = protein molecular weight marker.

The purified protein was constituted of 177 amino acids and full length (534 bp) PeBb1 sequence was cloned and transformed into pET-28a (+) vector, expressed in *E. coli* BL21 (DE3) (Figure 2). By using column chromatography, the recombinant protein was purified and desalted. Thereafter, we ran SDS-PAGE gel of purified recombinant protein and the results showed a single band of 19 kDa molecular weight (Figure 3). This was consistent with the calculated size of the fusion elicitor.

### 2.3. PeBb1-Induced Necrosis in Tobacco Leaves

Infiltration of PeBb1 into the leaves of tobacco ensued in quick macroscopic changes. There were conspicuous necrotic zones at the infiltration area recorded at 24 h post infiltration, whereas no necrosis was observed on the buffer infiltrated leaves (Figure 1C). It was observed that protein PeBb1 was stable and maintained its elicitor type activity of inducing necrotic lesions for 15 min at 4 °C or 25 °C, while it got denatured at 50, 75, and 100 °C (Supplementary Figure S1). Similarly, PeBb1 necrosis-inducing activity was observed only at a suitable pH range. At incubation for overnight in different pH solutions (pH 4, 6, 8, and 10) at 4 °C, necrotic reaction of PeBb1 was observed only at pH 6 or 8. 

### 2.4. Effect of PeBb1 Elicitor on the Fecundity Rate of M. persicae

**Figure 4:**
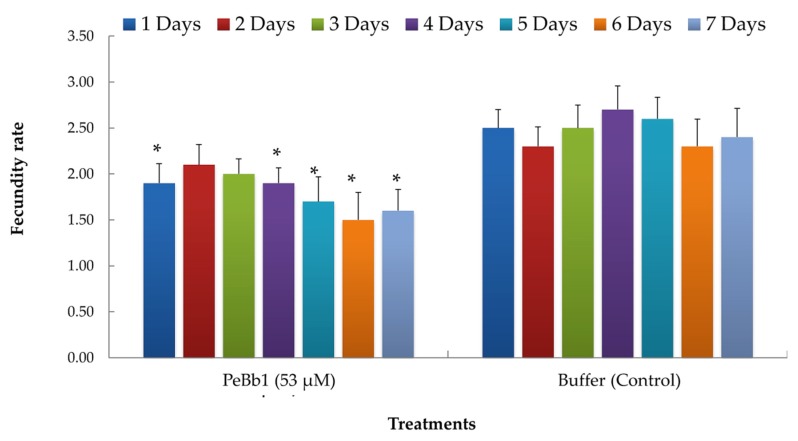
Mean fecundity of *M. persicae* recorded for the highest concentration of elicitor PeBb1 and for the control treatment. The columns represent mean fecundity rate ± SE (n = 10). Asterisk symbols indicate significant difference among the control and protein treatments (Student’s *t*-test at *p* ≤ 0.05).

Results of bioassay with PeBb1 elicitor protein showed a significant reduction in mean aphid fecundity as compared to the control (F_3,252_ = 9.58, p < 0.001), while the interaction of fecundity with time and time alone exhibited no significant effect (Table 1). In the control treatments, mean fecundity rate of aphids was 2.5 nymph^-1^ day^-1^ female^-1^. Among protein concentrations, minimum mean fecundity rate (1.7 nymph^-1^ day^-1^ female^-1^) was recorded for the highest concentration (53 μM) and it was significantly different from the control treatment (Figure 4). However, other two concentrations (i.e., 26 and 35 μM) showed a mean fecundity rate of 2.1 nymph^-1^ day^-1^ female^-1^ without any significant difference from the control treatment.

### 2.5. Expression of Plant Defense-Related Genes in Response to PeBb1 Elicitor

**Figure 5:**
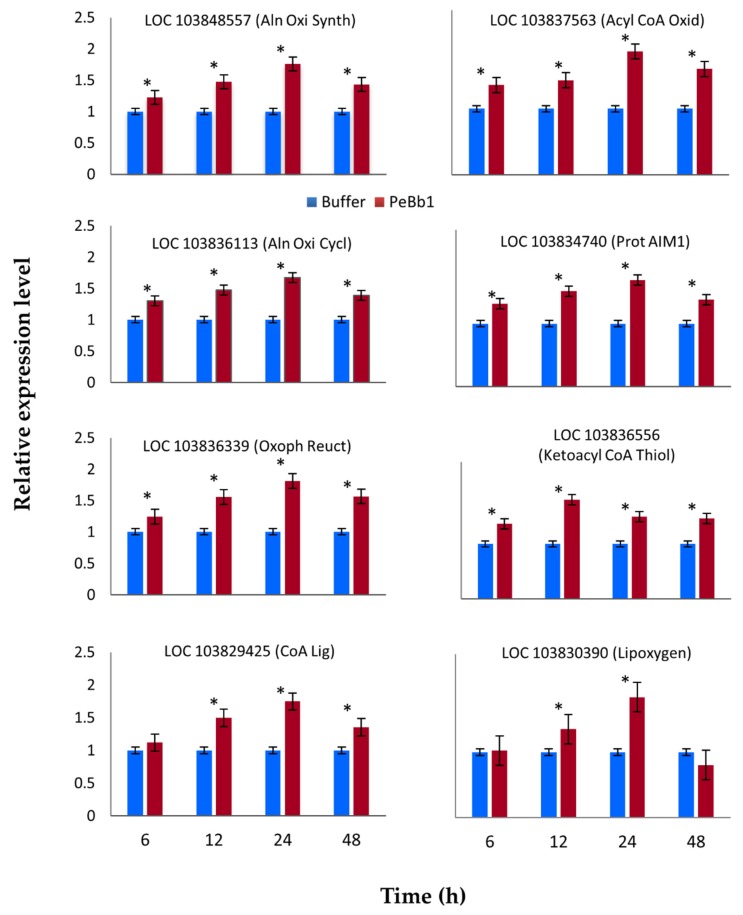
Relative expression levels of jasmonic acid (JA) pathway-related key genes determined at different time intervals post PeBb1 elicitor application. Blue and red columns show the result of buffer-treated (control) and elicitor-treated plants, respectively. For each gene, asterisk symbols show the significant difference among treatments (Student’s *t*-test at *p* ≤ 0.05).

**Figure 6:**
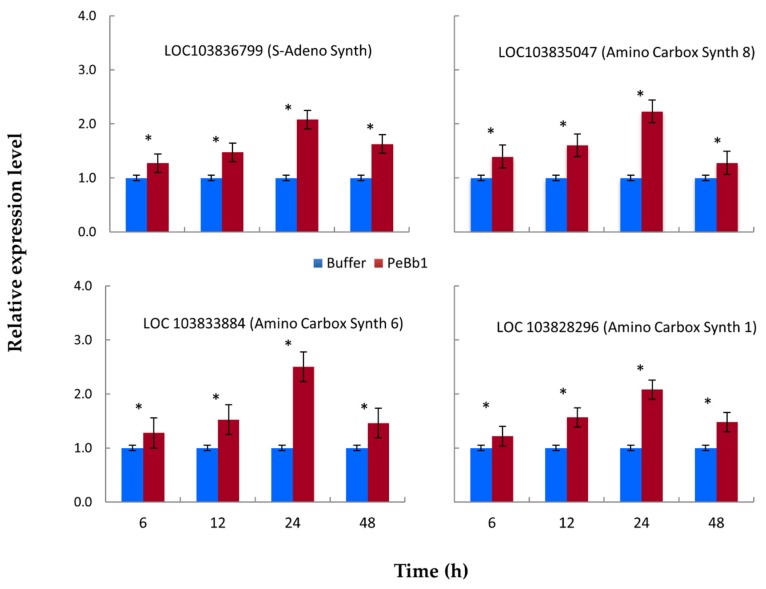
Relative expression levels of ethylene (ET) pathway-related key genes determined at different time intervals post PeBb1 elicitor application. Blue and red columns show the result of buffer-treated (control) and elicitor-treated plants, respectively. For each gene, asterisk symbols show the significant difference among treatments (Student’s *t*-test at *p* ≤ 0.05).

To clarify one of the putative modes of PeBb1 action resulting in plant resistance, we evaluated the expression profiles of ET and JA pathway related marker genes inducing plant defense mechanisms against aphids up to 48 h after treatment with PeBb1 (53 µM) or buffer. RT-qPCR analyses showed that JA pathway-associated genes were moderately elevated, while ET pathway-associated genes exhibited highest levels of expression. Almost all the JA pathway associated key genes (i.e., LOC103848557, LOC103836113, LOC103836339, LOC103829425, LOC103837563, LOC103834740 and LOC103836556) were up-regulated except one gene LOC103830390 that exhibited no considerable difference between the protein- and buffer-treated plants after 6 h of aphid attack and was down-regulated after 24 h of aphid attack (Figure 5). Maximum gene expression level was observed at 12 and 24 h post aphid infestation. On the other hand, all ethylene pathway linked genes (i.e., LOC103836799, LOC103835047, LOC103833884, and LOC103828296) were up-regulated at each time after the application of elicitor and aphid attack (Figure 6). 

## 3. Discussion

Elicitor proteins perform an important function in signaling plant defense pathways and are being considered as novel biological pest management strategies. Many biotrophic and necrotrophic microorganisms including pathogenic fungi are the main sources of different microbial elicitors (PAMPs or MAMPs) [33]. This study comprised of purification, molecular characterization, and in vitro evaluation of a yet uncharacterized elicitor protein PeBb1 derived from *B. bassiana* for its putative role against *M. persicae* (green peach aphids). We purified and cloned a 19-kDa protein (PeBb1) from *B. bassiana* ARSEF 2860 strain, having the ability to induce necrosis in tobacco plants and to reduce the fecundity of green peach aphid *M. persicae* on Chinese cabbage *B. rapa* plants. Moreover, this PeBb1 protein was able to up-regulate the transitory or local expression of ET and JA pathways associated plant defense genes in aphid infested elicitor-treated plants.

Our results demonstrated that aphid individuals developed significantly slower on the elicitor-treated *B. rapa* plants as compared to control plants because the exogenous application of PeBb1 elicitor protein considerably reduced the fecundity rate of *M. persicae* on *B. rapa* plants as compared to control ones, suggesting the putative role of PeBb1 in induced systemic resistance in *B. rapa* against *M. persicae*. These results are in accordance to some previous works indicating the detrimental effects of exogenous applications of different elicitor molecules such as benzothiadiazole (BTH), JA, and methyl jasmonate (MJ) on the fitness traits and population growth of aphids [34,35]. Nevertheless, some studies have shown how elicitors induce resistance against different sucking and chewing insect pests by the use of co-expression of different proteinase inhibitors [33,36,37]. Likewise, the exogenous treatment of different plant defense related proteins such as polyphenol oxidase and proteinase inhibitors have been shown to significantly reduce the incidence of insect pests on tomato plants [34,38]. 

JA, SA, and ET pathways play a significant role in inducing plant resistance against insects. Results of this study are in line with a recent work by Basit et al. [39] who revealed that different concentrations of an elicitor protein PeBC1, derived from a necrotrophic fungus *Botrytis cinerea*, reduced the fecundity rate of *M. persicae* concomitantly with a significant up-regulation of the expression of different SA and JA pathways-linked genes in common beans (*Phaseolus vulgaris*). These plant defense pathways are involved in the signaling transduction and regulation of downstream plant defense genes, thereby instigating a more effective plant defense response against insect pests [39,40,41]. Feeding by aphids induce both local and systemic defense reactions in plants [42]. Our findings showed the local expression of ET and JA responsive genes in *B. rapa* leaves, though no systemic change in the expression levels of these genes has been observed. PeBb1 induced a significant and strong up-regulation of the expression of all ET and JA pathway related genes.

## 4. Materials and Methods

### 4.1. Rearing of Aphids

Individual clones of *M. persicae* (green peach aphid) were collected from the young seedlings of Chinese cabbage (*Brassica rapa* ssp. *pekinensis*) maintained in the greenhouse facility of Chinese Academy of Agricultural Sciences (CAAS), Beijing, China. Aphids were reared on the same plant species at 25 ± 2 °C temperature and 50–60% relative humidity under 16:8 h light: dark photoperiod. During the entire study period, plants were changed every week ^33^.

### 4.2. Plant, Pathogen and Bacterial Culture

Seeds of *B. rapa* ssp. *pekinensis* were allowed to germinate in a 9-cm Petri dish for 72 h at room temperature (27 °C) and then were transplanted to pots containing sterilized soil mixture in a growth chamber at 25 ± 2 °C temperature and 60% relative humidity under 16:8 h light:dark photoperiod. Similarly, plants of tobacco (*Nicotiana tabacum* cv. Samsun-NN) were raised in a growth chamber at 25 ± 2°C under a 16:8 h light:dark photoperiod. Fungal isolate of *Beauveria bassiana* strain ARSEF 2860 (BB-72) was maintained on solid media of potato dextrose agar (PDA: 20 g L^−1^ agar, 20 g L^−1^ dextrose and 200 g L^−1^ potato) and LBA agar (5 g L^−1^ yeast extract, 10 g L^−1^ tryptone and NaCl each and 15 g L^−1^ agar). Liquid medium used for bacterial culture was Luria-Bertani (LB) broth.

### 4.3. Isolation of Crude Protein

For primary culture of *B. bassiana* strain ARSEF 2860 (BB-72), 1 mL of conidial suspension (1.0 × 10^8^ conidia mL^−1^) was added in 25 mL of Adámek’s liquid medium (40 g yeast extract (Difco, Detroit, MI, USA), 40 g dextrose and 30 g corn steep liquor (Sigma-Aldrich, Saint Louis, MO, USA)). Incubation of primary culture was done in a rotary shaker at 200 rpm at 25 °C for three days. Thereafter, secondary culture was made by accumulation of 10 mL primary culture into 1 L of Adámek’s liquid medium for six days at 140 rpm at 25 °C. The sample was centrifuged for 20 min at 12000 rpm at 4 °C, and the supernatant was filtered with a 0.45 μm pore size filter (Millipore Corp., Billerica, MA, USA) to acquire the filtrate. Ammonium sulfate (NH)_4_SO_4_ was added to fungal filtrate to attain 80% (*w/v*) relative saturation at 4 °C overnight and the complex was centrifuged for 15 min at 12000 rpm at 4 °C. The precipitate was dissolved in 30 mL buffer A (50 mM Tris-HCL, pH 8.0) and was dialyzed against the buffer A for 48 h to remove (NH)_4_SO_4_. The insoluble debris was removed from the dialysate by centrifugation for 15 min at 12000 rpm at 44 °C, and then the crude protein was filtered with a 0.22 μm pore size filter (Millipore, Corp., Billerica, MA, USA). The extracted crude protein was stored at -80 °C till further purification. The crude protein (50 μL) was analyzed for its potential to elicitate necrosis in tobacco plants.

### 4.4. Protein Purification and Mass Spectrometry

Purification was carried out with the ÄKTA Explorer 10 protein purification system (GE Healthcare, Piscataway, NJ, USA). The crude protein was loaded on an anion exchange chromatography column (HP Q HiTrap^TM^ 5 mL, GE Healthcare, Uppsala, Sweden), previously equilibrated with buffer A (50 mM Tris-HCL, pH 8.0). The bounded proteins were eluted with buffer B (50 mM Tris-HCL, 1.0 M NaCl, pH 8.0) at a flow rate of 2 mL min^−1^ and then all fractions were collected. Each fraction was applied to a desalting column (GE Healthcare, Uppsala, Sweden) and was observed for its anti-insect activity against *M. persicae*. Moreover, purified *B. bassiana*-derived protein was also tested for its ability to induce necrosis in the leaves of tobacco. The fraction that showed maximum necrosis induction was further purified using 12% sodium dodecyl sulfate polyacrylamide gel electrophoresis (SDS-PAGE) gel and was tested for necrosis-inducing potential of the purified protein fraction. The fractions that showed activity were collected and concentrated by ultrafiltration and were washed 3 times with buffer A (50 mM Tris-HCL, pH 8.0) and were stored at -80 °C. Protein sample isolated on SDS-PAGE gel was further characterized by mass spectrometry (MS) analysis (Beijing Protein Innovation Co. Ltd., Beijing, China). Using MASCOT search engine (Matrix Science Inc., London, UK; http://www.matrixscience.com), the tandem MS (MS-MS) data were analyzed automatically.

### 4.5. Gene Cloning

Using E.Z.N.A.^®^ fungal RNA Kit (Omega Bio-Tek Inc., Norcross, GA, USA), total RNA was extracted from the fungal cells. First-strand cDNA was synthesized using TransScript^®^ One-Step gDNA Removal and cDNA Synthesis Super Mix Kit (TransGen Biotech, Beijing, China). On the basis of peptide sequence retrieved from the National Center for Biotechnology Information (NCBI) database and the *de novo* sequencing acquired by MS analysis, a pair of gene-specific primers (forward primer: 5′-ATGCAAGATGCGTTGCCAGAG-3′ and reverse primer: 5′-TCAGCCATAATGGACACATTGAC-3′) was designed to amplify the entire coding sequence of elicitor protein-encoding gene of *B. bassiana*. Primer pairs were designed based on the sequence of XM_008605366 gene encoding protein of XP_008603588.1.

The amplified gene was cloned into the pET-28a(+) vectors (Novagen, USA) and was transformed into the competent cells of *Escherichia coli* BL21 (DE3) (TransGen Biotech, Beijing, China), and then was cultured in LB broth in a shaker for 14 h. The cells were harvested by centrifugation and the plasmids were extracted from these harvested cells. The full-length cDNA of PeBb1 gene was ratified by DNA sequencing (Beijing Genomics Institution, Beijing, China).

### 4.6. Expression and Purification of Recombinant Protein

In order to express the elicitor as a C-terminally His6-tagged protein, the PeBb1 gene was inserted into the *Bam*HI/*Hind*III sites of His-tagging pET-28a(+) vector (Novagen, San Diego, CA, USA) and was then transformed into the competent cells of *E. coli* strain BL21 (DE3) (TransGen Biotech, Beijing, China). The primers designed included the *BamH1/HindIII* restriction sites and the 5′ and 3′ ends of the PeBb1 gene (forward primer: 5′-GGATCCATGCAAGATGCGTTGCCAGAG-3′ and reverse primer: 5′-AAGCTTTCAGCCATAATGGACACATTGAC-3′ (restriction sites are underlined). The clones of PeBb1 gene insertion were identified by PCR. The PCR thermal protocol was as follows; 95 °C for 5 min, followed by 35 cycles of 95 °C for 30 s, 62 °C for 30 s, and 72 °C for 30 s and a final extension at 72 °C for 10 min. Amplified DNA was isolated on a 1% agarose gel via gel electrophoresis and was detected visually by staining with Gold View (SBS Genetech, Beijing, China) using Trans2K^®^ Plus II DNA Marker (TransGen Biotech, Beijing, China).

For the expression of recombinant PeBb1 protein, bacteria were cultured for 4 h at 37 °C, and then 200 μM isopropyl β-D-1-thiogalactopyranoside (Sigma, St. Louis, MO, USA) was added to the culture when OD_600_ was 0.6–0.8, to subsequently induce the recombinant protein for 14–16 h at 200 rpm and at 16 °C. Bacterial cells were obtained by centrifugation and then were re-suspended in buffer C (50 mM Tris-HCl, 200 mM NaCl, pH 8.0) and the cells were disrupted by ultrasonic disruptor for three times. Supernatant having the recombinant protein was collected by centrifugation for 30 min at 12000 rpm. PeBb1 protein was further purified by affinity chromatography through a His-Trap HP column (GE Healthcare, Waukesha, WI, USA) using loading buffer C. It was directly eluted with buffer D (50 mM Tris-HCl, 200 mM NaCl, 500 mM imidazole, pH 8.3) and was subsequently desalted with buffer E (50 mM Tris-HCl, pH 8.3) in a HiTrap^TM^ desalting column (GE Healthcare, Waukesha, WI, USA). The molecular weight was examined in a 12% SDS-PAGE gel and a protein marker (Thermo Scientific, Rockford, IL, USA) was used to assess the apparent molecular weight of the purified recombinant elicitor protein.

### 4.7. Characterization of PeBb1 Elicitor Protein

Following the protein purification, the necrosis-inducing activity of protein was assayed in 8-week old plants of tobacco (*N. tabacum* cv. Samsun-NN). For this purpose, mesophyll tissues of completely developed leaves were infiltrated using mock buffer (50 µL of 50 mM Tris-HCL, pH 8.0) with the help of a syringe (without needle) covering an area of 1 cm^2^. The necrosis symptoms were precisely examined after 24 h according to the method described by D’Silva and Heath [43]. To check the heat stability and pH of the elicitor, the recombinant PeBb1 was treated at different temperatures (i.e., 4, 25, 50, 75 and 100 ℃) for 15 min and also at different pH values (i.e., 4, 6, 8 and 10) overnight. The necrosis induction by the elicitor protein was observed after 24 h.

### 4.8. Bioassay of Elicitor Activity against M. Persicae

The laboratory bioassay was carried out to assess the activity of elicitor PeBb1 against *M. persicae* (green peach aphid). Treatments included three concentrations of the elicitor protein (i.e., 26, 35, and 53 μM) and one control (buffer A; 50 mM Tris-HCl; pH 8.0). All protein concentrations were measured by BCA Protein Assay Kit (Pierce, Rockford, IL, USA). For bioassay, potted Chinese cabbage (*B. rapa*) plants at 4-leaf stage were used. Each plant was sprayed by approximately 4 mL of the elicitor PeBb1 solution with the help of an aerosol spray bottle, and was placed for 24 h to dry. Ten freshly moulted (0 to 6 h old) adult apterous aphids were confined for 12 h on each treated cabbage plant, and then all aphid individuals were removed, while leaving 10 active and healthy aphid nymphs each plant. In order to determine the fecundity, one survived adult aphid per leaf was confined using a small leaf cage. Data regarding number of offspring per adult aphid was noted for one week. Ten replications were maintained for each treatment. All treatments were maintained at 50 to 60% relative humidity and 25 ± 2 °C temperature.

### 4.9. Expression Analysis of Plant Defense-Related Genes Using RT-qPCR

To assess the plant defense mechanisms induced by the exogenous foliar application of PeBb1 elicitor on *B. rapa* plants, relative expression of key genes associated with *B. rapa*’s ET and JA pathways (Table 2) were examined by real-time quantitative PCR (RT-qPCR). Using the EasyPure^®^ Plant RNA Kit (TransGen Biotech, Beijing, China), total RNA was extracted from the leaves of aphid infested protein-treated and buffer-treated (control) plants of *B. rapa*. TransScript^®^ All-in-One SuperMix for qPCR Kit (TransGen Biotech, Beijing, China) was used to synthesize first-strand cDNA. The relative expression levels of both type of defense-related genes were determined by RT-qPCR performed on ABI 7500 Real-Time PCR System thermocycler (Applied Biosystems, Foster City, CA, USA) using TransStart^®^ Green qPCR SuperMix UDG (TransGen Biotech, Beijing, China). Three independent biological and three technical replicates were performed for each sample. *Actin* was used as a quantitative control. Relative expression levels were calculated using the 2^–ΔΔCT^ method [44]. Primer pairs used for the amplification of these plant defense associated genes are detailed in Table 3.

### 4.10. Statistical Analysis

All experiments were conducted in three independent replications and the data are presented as the means along with standard errors. Using SAS^®^ Statistical Software Program (SAS Institute Inc., Cary, NC, USA), significant differences between the treatments (elicitor concentrations) were calculated by one-way factorial analysis of variance (ANOVA) and Fisher’s least significant difference (LSD) test was used for pairwise comparisons at the probability level of 0.05. Comparative CT (2^–ΔΔCT^) method was used to determine the RT-qPCR expression levels. Moreover, data of elicitor-treated and buffer-treated (control) plants were compared using the Student’s *t*-test at the probability level of 0.05.

## 5. Conclusions

This study reports the purification, characterization, and evaluation of a yet uncharacterized protein elicitor PeBb1 from *B. bassiana* strain ARSEF 2860 as putative microbial pest control tool against green peach aphid *M. persicae.* The bioassays with recombinant PeBb1 protein showed a significant reduction in mean aphid fecundity and a significant up-regulation of the expression levels of ET and JA pathway related genes in the protein-treated *B. rapa* ssp. *pekinensis* plants. These findings suggest that PeBb1 can be a prospective candidate molecule to enhance the defense mechanism in *B. rapa* ssp. *pekinensis*. Hence, activating plant defense responses with PeBb1 elicitor protein, an alternative method like biopesticides and transgenic crops, can be exploited to control the most destructive pest *M. persicae* and to reduce the risk of environmental contamination with synthetic chemical insecticides. Our research presents the potential of a yet uncharacterized elicitor protein from fungus *B. bassiana* to fortify host plant resistance against pests. However, additional studies are needed to better understand the processes by which the changes are induced in *B. rapa* by the elicitor PeBb1 and to find out how exactly these responses influence the fitness traits such as fecundity of aphids *M. persicae*, and to see if this protein is produced by other strains of *B. bassiana* and elicits similar defense response in other plant species. Moreover, assessing sublethal effects of PeBb1 elicitor on other life-history traits of aphids, such as nymphal development time, adult longevity, mortality, intrinsic rate of population increase etc., constitutes the future perspective of the study.

## Figures and Tables

**Table 1:** Factorial analysis of variance for the effect of elicitor protein PeBb1 extracted from *B. bassiana* ARSEF 2860 strain on the fecundity rate of *M. persicae*.

SOV	DF	SS	MS	F-Value	*p*-Value
Concentration	3	18.67	6.224	9.58	<0.001
Time	6	3.94	0.656	1.01	0.4198
Concentration × Time	18	3.18	0.177	0.27	0.9989
Error	252	163.80	0.650		
Total	279	189.586			
GM/CV	2.19/36.77				

*p* < 0.05 = significant and *p* < 0.001 = highly significant; one-way factorial ANOVA at α = 0.05; MS = mean sum of squares; SS = sum of squares; DF = degree of freedom; F = F-statistic; CV = coefficient of variation; GM = grand mean.

**Table 2:** Key genes associated with the ethylene (ET) and jasmonic (JA) pathways of *B. rapa* ssp. *pekinensis*.

Sr. No.	Gene ID	Gene Biochemical Name (Abbreviated)	Gene Biochemical Name (Detailed)
Jasmonic Pathway			
1	LOC103848557	Aln Oxi Synth	Allene oxide synthase, chloroplastic
2	LOC103836113	Aln Oxi Cycl	Allene oxide cyclase 4, chloroplastic
3	LOC103836339	Oxoph Reuct	Putative 12-oxophytodienoate reductase-like protein 1
4	LOC103829425	CoA Lig	4-coumarate--CoA ligase-like 4
5	LOC103837563	Acyl CoA Oxid	peroxisomal-like Acyl-coenzyme A oxidase 2
6	LOC103834740	Prot AIM1	Peroxisomal fatty acid beta-oxidation multifunctional protein AIM1-like
7	LOC103836556	Ketoacyl CoA Thiol	Peroxisomal-like 3-ketoacyl-CoA thiolase
8	LOC103830390	Lipoxygen	Lipoxygenase 2, chloroplastic-like
Ethylene Pathway			
1	LOC103836799	S-Adeno Synth	S-adenosylmethionine synthase-like
2	LOC103835047	Amino Carbox Synth 8	1-aminocyclopropane-1-carboxylate synthase 8
3	LOC103833884	Amino Carbox Synth 6	1-aminocyclopropane-1-carboxylate synthase 6
4	LOC103828296	Amino Carbox Synth 1	1-aminocyclopropane-1-carboxylate oxidase 1

**Table 3:** Primer sequences used for RT-qPCR amplifications of the defense-related and quantitative control genes of *B. rapa* spp. *pekinensis.*

Genes	Forward Primer (5′-3′)	Reverse Primer (5′-3′)
Actin	TATGCTCTTCCACATGCTATTC	CCTTACGATTTCACGCTCTG
103848557	TTACTTCCACAAGCAAAAACCC	TTATCAACATCGAACAAAACCG
103836113	CGACCTCGTCCCTTTCACTA	AGCGTTCGCCTTTCTTCTCA
103836339	TCAGAACACTCTATTGCCACAT	CCTTCAAACGCCTTCCTCAT
103829425	CTATGGGCTACTCTGCTTCACT	CTCCGTCACCTCCTTACTCAA
103837563	CCAACGCACGACACCAAAGG	GAACGGAACCGCAAAGCCCC
103834740	CCTCTTTCGGCTTGCCATTA	TCCCATTTCTCCCGCTTTTA
103836556	GCTTCATCATCTTCAACCTC	CTTCTCTATCACCGCTCTCA
103830390	GGTCTTCACGCCAGGTTATG	ATTGTCTGTTTGCCGCTATT
103836799	GCAAAGTCCTCGTCAACATC	TCATCAGTAGCGTACCCAAA
103835047	CCTGGAGATGCTTTCTTGCT	TTAGTTCGGTTCGGGTTGTT
103833884	CATCCGCAAGAGCAAACTAC	CCATCCATATGAACAAACCG
103828296	GTGAAAATCTTGGTCTCCCTCG	CAGTATGTTCTCTCAGCCCTCT

## Supplementary Materials

The following are available online at https://www.mdpi.com/2076-0817/9/2/111/s1, Figure S1: The hypersensitive response induced by elicitor protein PeBb1 (53 µM L^−1^) in leaves of tobacco (*Nicotiana tabacum* cv. Samsun-NN) at different temperature regimes captured 24 h post infiltration.
