# Supplementary material for: Putative Role of a Yet Uncharacterized Protein Elicitor PeBb1 Derived from Beauveria bassiana ARSEF 2860 Strain against Myzus persicae (Homoptera: Aphididae) in Brassica rapa ssp. pekinensis
Source: Pathogens. 2020 Feb 11;9(2):111. doi: 10.3390/pathogens9020111 (PMC7167858; doi:10.3390/pathogens9020111)
Supplement: Supplementary file 1 [file pathogens-09-00111-s001.zip › Supplementary Figure S1.docx]

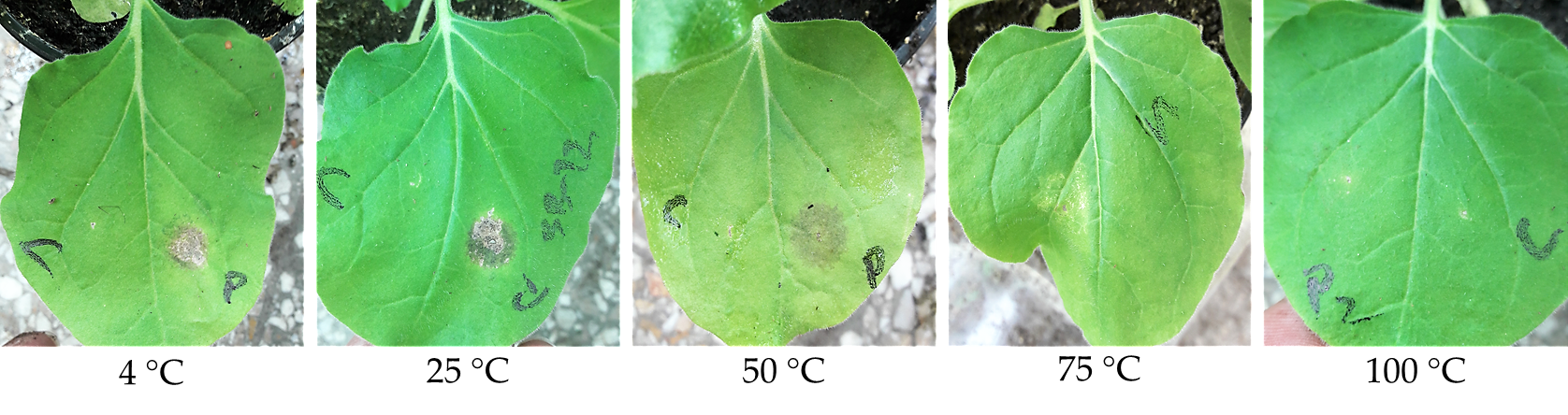
**Supplementary Figure S1:** The hypersensitive response induced by elicitor protein PeBb1 (53 µM L^-1^) in leaves of tobacco (*Nicotiana tabacum* cv. Samsun-NN) at different temperature regimes captured 24 h post infiltration.
